# Malignant infantile osteopetrosis: case report with review of literature

**DOI:** 10.11604/pamj.2014.17.63.3759

**Published:** 2014-01-27

**Authors:** Laila Essabar, Toufik Meskini, Said Ettair, Naima Erreimi, Nezha Mouane

**Affiliations:** 1 Department of Pediatric Hepatology Gastroenterology and Nutrition – P III – Rabat Children's Hospital, Rabat, Morocco

**Keywords:** Osteopetrosis, infant, hepatomegaly, splenomegaly, hydrocephalus, rickets

## Abstract

Malignant Infantile Osteopetrosis (MIOP) is a rare genetic disorder due to osteoclast abnormal activity. We report a thirteen month-old male patient, diagnosed as MIOP while investigating the cause of hepatosplenomegaly associated with hydrocephalus. His medical history revealed non consanguineous parents and one brother's death at the same age of unknown etiology (similar symptoms). Systemic examination showed hepatosplenomegaly, growth failure, developmental milestones delay, and rickets features. Ophthalmic exam yielded bilateral optic atrophy. Skeleton radiographs detected generalized dense bone and rickets. Cerebral CT scan revealed hydrocephalus. Histological examination showed hypoplastic bone marrow and extra-medullary hematopoeisis. Diagnosis was confirmed by genetic testing that showed two heterozygote mutations within the TCIRG1 gene. The patient received supportive treatment. He died from an acute respiratory distress. MIOP should be kept in mind as a rare cause of hepatosplenomegaly. Early diagnosis and timely Hematopoietic stem cell transplantation are the only curative approach for an otherwise fatal disease.

## Introduction

Malignant infantile osteopetrosis (MIOP) is a rare genetic disorder that is characterised by increased bone density due to abnormal osteoclast activity. Consequent impaired bone resoption and endochondral formation replaces haematopoietic cells in the medullary cavity, and also increases the incidence of fractures due to bone fragility. The reduction in haematopoietic cells can also cause haematological abnormalities including thrombocytopenia, anaemia, susceptibility to infections and extramedullary haematopoiesis. Neurological manifestations of osteopetrosis can also occur due to narrowing of osseous foramina. We report a case of MIOP with numerous complications including hydrocephalus and rickets.

## Patient and observation

A thirteen month-old male patient was referred to department of pediatric gastroenterology of Rabat Children's Hospital with the chief complaint of hepatosplenomegaly associated with hydrocephalus. He was born at term by normal vaginal delivery without complications and his birth weight was 3200g. Parents were non consanguineous, one brother died at the same age of unknown etiology (with similar symptoms). According to his past medical history, spleen and liver enlargement was first observed at the age of four months by a physician, blood investigations were performed. The patient was lost to follow-up until the age of 13 months when parents consulted a pediatrician for macrocephaly and abdominal overdistension. The patient was referred to our department for etiological investigations of hepatosplenomegaly and hydrocephalus.

On general physical examination, the patient was short statured (his length and weight were both < 3^rd^ percentile), his cranial perimeter was between the 75th ‘ 90th percentiles with a bulging anterior fontanel. Delayed physical developmental milestones were noted. Patient was pale and was not icteric. Abdomen was distended with hepatomegaly with the liver noted to be 4cm and splenomegaly with the spleen noted to be 7cm. Intraoral examination showed delayed tooth eruption. Skeletal exam revealed frontal bossing, palpable costochondral beading and bowlegs. Ophtalmic exam showed convergent squint with loss of visual fixation and pursuit, fundoscopic examination disclosed bilateral optic atrophy. Blood investigations revealed microcytic, hypochromic anemia with hemoglobin 9.4 g/dl; white blood cell count 20800 cells/mm^3^; platelet count 130.000/mm^3^.

Biochemical analysis showed the following abnormalities: calcaemia 76mg/l; alkaline phosphatase 328 IU/l; parathyroid hormone 135 pg/ml. Serology for toxoplasma, hepatitis virus, cytomegalovirus and rubella was negative. Immunoglobulin (G, A, M) levels were normal.

**Figure 1:**
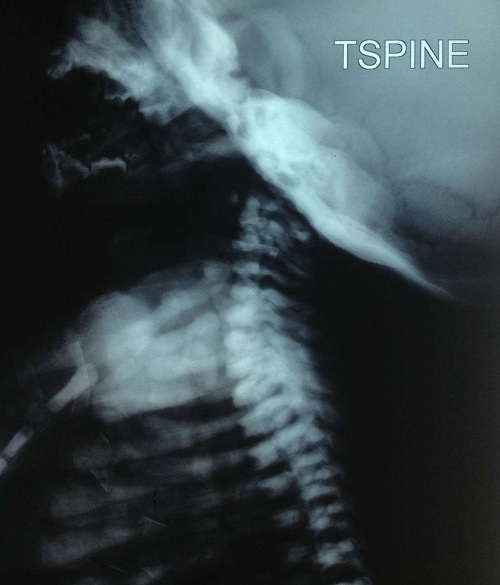
Lateral skull radiograph. Note increased thickness of skull base

**Figure 2:**
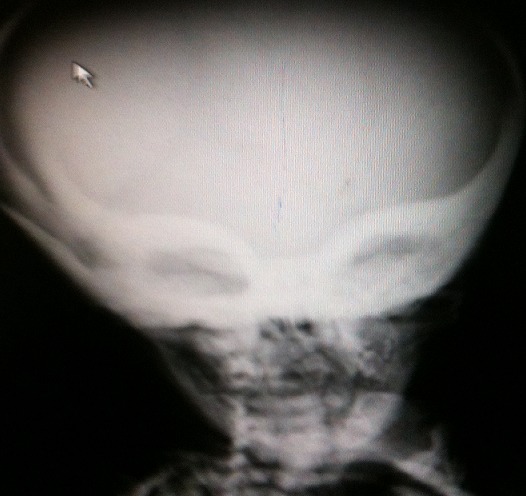
Facial skull radiograph. Note sclerosis of the orbits and sphenoid bones resulting in “Harlequin mask appearance’’

**Figure 3:**
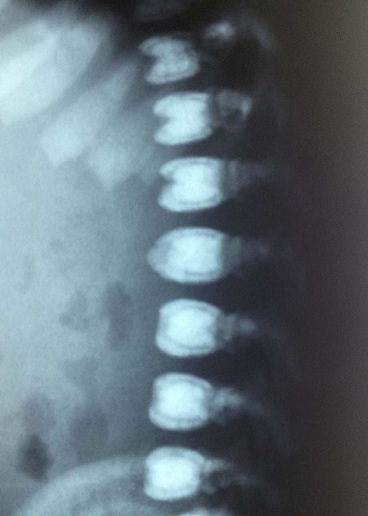
Lateral spine radiograph. Note vertebral sclerosis resulting in “sandwich vertebrae appearance’’

**Figure 4:**
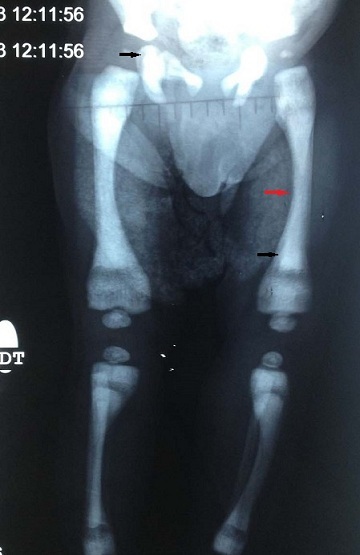
Pelvis and lower limbs radiograph. Note generalized bone density with bone in bone appearance (black arrows) and bowlegs (red arrow)

**Figure 5:**
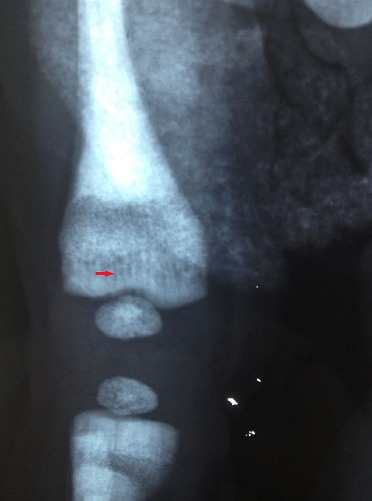
Right femur radiograph. Note metaphyseal modelling defects and characteristic lucent bands (arrow)

**Figure 6:**
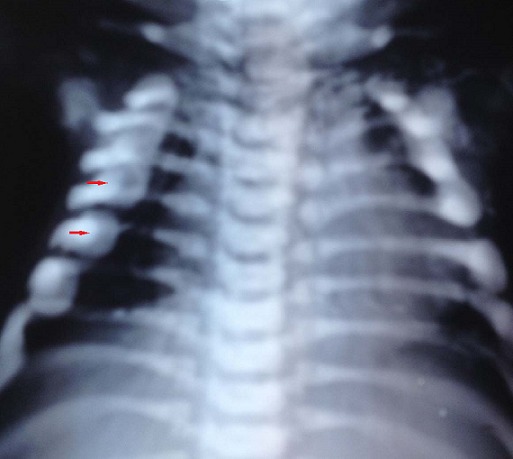
Chest radiograph. Note rachitic rosary

Chest, skull and vertebrae radiographs detected generalized dense bone particularly in the skull base (Figure 1, Figure 2) and the vertebrae with “bone-in-bone” appearance (Figure 3, Figure 4) and bone modelling affecting the metaphyses of long bones (Figure 5), radiographic features of superimposed rickets was also noted(Figure 5, Figure 6). Abdominal ultrasonography disclosed pronounced hepatosplenomegaly. CT scan revealed triventricular hydrocephalus due to aqueductal stenosis. Bone marrow aspiration showed hypocellularity. Osteo-medullary biopsy demonstrated hypoplastic bone marrow, rare osteoclasts were noted. Liver biopsy revealed extra-medullary hematopoeisis with ballooned and clarified hepatocytes.

Storage disease (such as mucopolysaccharidosis) and congenital infectious disorders (TORCH) were considered in the differential diagnosis. Urine Ph was alkaline, metabolic acidosis and intracerebral calcifications were absent, therefore carbonic anhydrase II deficiency syndrome was eliminated.

However, correlating the radiographic features with the clinical features, the case was diagnosed as infantile malignant OP. Diagnosis was confirmed by genetic testing which revealed two heterozygote mutations (c. 196 + 1G > T); (2236 + 1G > A) within the TCIRG1 gene.

The patient received symptomatic and supportive treatment including antibiotic therapy, rhinopharyngeal disinfection, respiratory kinesitherapy, calcium and vitamin D supplementation. Patient was advised for bone marrow transplantation (BMT) which was not done due to cost. Patient was finally referred for ventriculoperitoneal shunt. He died from an acute respiratory distress.

## Discussion

Osteopetrosis is clinically a highly heterogeneous group of conditions that share the hallmark of increased bone density on radiographs due to abnormalities in osteoclast differentiation or function [1]. There are four subtypes of OP (a) malignant or infantile OP, (b) Benign or adult OP, (c) intermediate OP, and (d) carbon anhydrase type II (CAII) deficiency [2].

Malignant infantile osteopetrosis (MIOP) is the autosomal recessively inherited form of this disease that generally begins in utero [3], it presents at birth [4, 5], or within the first year of life and is associated with increased severity compared to the autosomal dominant form [6]. Our patient had the symptoms since the age of four months. It has an incidence of 1 in 250.000 births, with a particularly high incidence reported in Costa Rica (3.4:100000) [7, 8].

The increase in bone mass leads to phenotypic features such as macrocephaly and frontal bossing. Tooth eruption defects are also common. The longitudinal growth of bones is impaired with a short stature and predisposition to fractures and osteomyelitis.

Our reported case showed all these characters except bony fractures and osteomyelitis.

The abnormal expansion of bone interferes with medullary haematopoiesis, resulting in life-threatening anemia, thrombocytopenia, increased susceptibility to infections, and secondary expansion of extramedullary haematopoiesis sites such as the liver and spleen. Osteomedullary biopsy of our patient disclosed bone marrow failure and liver biopsy demonstrated extramedullary hematopoeisis.

The most commonly observed neurological manifestations of osteopetrosis are secondary to obstruction of the foramina through which the cranial nerves, spinal cord and major blood vessels transverse the skull, resulting in blindness, hearing loss, facial palsy and hydrocephalus [9, 10]. Distinct from these compressive phenomena, some patients with autosomal recessive osteopetrosis variants (neuropathic ARO) display signs of primary neurodegeneration including primary seizures in the setting of normal calcium levels, developmental delay, hypotonia, retinal atrophy and sensorineural deafness[9]. Reported brain MRI findings include significantly delayed myelination and diffuse progressive cortical and subcortical atrophy [11, 12]. Children with MIOP are at risk of developing hypocalcaemia, with attendant tetanic seizures and secondary hyperparathyroidism[1 4, 5]. Rickets has been also observed as a complication of MIOP [13]. The above patient showed hypocalcaemia and rickets.

Characteristic radiographic findings in osteopetrosis include a marked increase in bone density with defective metaphyseal remodeling, and a “bone within a bone” appearance [7]. Alternating sclerotic and lucent bands can give the vertebrae a ‘sandwich’ appearance. Computerized tomography scan can be used for diagnosis and to determine the effect of the treatment. It is also used to assess auditory and optic canal [14]. The skeletal survey and CT scan of our patient was specific for radiologic findings of osteopetrosis.

Genetic testing can be used to confirm the diagnosis and differentiate between different subtypes of osteopetrosis, providing additional information regarding prognosis, likely response to treatment and recurrence risks [1]. Our patient's genetic analysis showed two heterozygote mutations of the TCIRG 1 gene; therefore diagnosis of autosomal recessive inherited osteopetrosis or MIOP was confirmed. Bone biopsy can distinguish between osteoclast-poor and osteoclast-rich subtypes of MIOP [1], osteomedullary biopsy of our patient revealed rare osteoclasts.

Primary sclerosing conditions of bone caused by osteoclast dysfunction need to be distinguished from the large number of conditions in which bone sclerosis can occur as a secondary phenomenon. Some alternative diagnosis to consider include pseudohypoparathyroidism, pyknodysostosis, and hypoparathyroidism, chemical poisoning (e.g., lead, fluoride, and beryllium), malignancies (myeloproliferative diseases and leukemia) [7].

In our case, besides the above-mentioned diagnosis, storage disease and congenital acquired infection were excluded because laboratory tests findings did not support them. Based on clinical history, radiographic findings and mutation analysis, our case was diagnosed as the infantile or malignant type, with autosomal recessive inheritance. Management of patients with osteopetrosis requires a comprehensive approach to characterstic clinical problems including hematological and metabolic abnormalities, recurrent infections, bone complications and neurological sequel [15].

At present, Hematopoietic stem cell transplantation (HSCT) offers the only chance of cure for MIOP; it should be performed early before the irreversible neurologic impairment. HSCT replaces abnormal osteoclasts with normal cells, given the high associated morbidity and mortality it's reserved only for the most severe cases of osteopetrosis [3]. Successful results have been achieved in patients transplanted with allogenic donor stem cells [16]. Furthermore, non-allogenic HSCT may be an option to treat MIOP, it showed high survival rate and restoration of hematopoiesis in haploid transplant patients [17].

In our case, HSCT was not performed given its high cost; therefore, treatment was largely supportive and was aimed at providing surveillance and symptomatic management of complications such as antibiotic therapy, calcium and vitamin D supplementation and nutritional measures. Surgical decompression of the optic nerve was also discussed; the patient was referred for ventriculoperitoneal shunt. He died at the age of fourteen months from an acute respiratory distress.

Genetic counseling is important. Antenatal diagnosis in families with MIOP may be possible using radiographs [18], thus allowing haematopoietic stem cell transplantation (HSCT) before the age of 3 months with the aim of improving neurological outcomes. However, the difficulty in obtaining conclusive results by radiographic evaluation of fetus in utero makes prenatal molecular diagnosis highly desirable [19]. Our patient's parents have benefited from a genetic consultation. Prenatal diagnosis is planned for the forthcoming pregnancies.

## Conclusion

Although diagnosis of MIOP is easy and depends mainly on radiographic examination, it's often delayed due to rarity of the disease and lack of clinical suspicion. Early diagnosis and timely HSCT is the only curative treatment approach for MIOP, an otherwise fatal disease.
